# Snacktivity™ to promote physical activity and reduce future risk of disease in the population: protocol for a feasibility randomised controlled trial and nested qualitative study

**DOI:** 10.1186/s40814-023-01272-8

**Published:** 2023-03-17

**Authors:** Amanda J. Daley, Ryan A. Griffin, Catherine A. Moakes, James P. Sanders, Magdalena Skrybant, Natalie Ives, Ben Maylor, Sheila M. Greenfield, Kajal Gokal, Helen M. Parretti, Stuart J. H. Biddle, Colin Greaves, Ralph Maddison, Nanette Mutrie, Dale W. Esliger, Lauren Sherar, Charlotte L. Edwardson, Tom Yates, Emma Frew, Sarah Tearne, Kate Jolly

**Affiliations:** 1 Centre for Lifestyle Medicine and Behaviour, School of Sport, Exercise and Health Sciences, Loughborough University, Loughborough, UK; 2 Birmingham Clinical Trials Unit, Institute for Applied Health Research, University of Birmingham, Birmingham, UK; 3 Institute for Applied Health Research, University of Birmingham, Birmingham, UK; 4 Diabetes Research Centre, College of Life Sciences, University of Leicester and NIHR Leicester Biomedical Research Centre, Leicester, UK; 5 Norwich Medical School, Faculty of Medicine and Health, University of East Anglia, Norwich, UK; 6 University of Southern Queensland, Springfield, Australia; 7 Faculty of Sport & Health Sciences, University of Jyväskylä, Jyväskylä, Finland; 8 School of Sport, Exercise and Rehabilitation Sciences, University of Birmingham, Birmingham, UK; 9 Institute for Physical Activity and Nutrition, Deakin University, Melbourne, Australia; 10 Physical Activity for Health Research Centre, University of Edinburgh, Edinburgh, UK; 11 School of Sport, Exercise and Health Sciences, Loughborough University, Loughborough, UK; 12 Health Economics Unit, Institute for Applied Health Research, University of Birmingham, Birmingham, UK

**Keywords:** Physical activity, Snacktivity™, Health, Randomised feasibility trial, Interviews, Small bouts

## Abstract

**Background:**

Many people do not regularly participate in physical activity, which may negatively impact their health. Current physical activity guidelines are focused on promoting weekly accumulation of at least 150 min of moderate to vigorous intensity physical activity (MVPA). Whilst revised guidance now recognises the importance of making small changes to physical activity behaviour, guidance still focuses on adults needing to achieve at least 150 min of MVPA per week. An alternative ‘whole day’ approach that could motivate the public to be more physically active, is a concept called Snacktivity™. Instead of focusing on achieving 150 min per week of physical activity, for example 30 min of MVPA over 5 days, Snacktivity™ encourages the public to achieve this through small, but frequent, 2–5 min ‘snacks’ of MVPA throughout the whole day.

**Methods:**

The primary aim is to undertake a feasibility trial with nested qualitative interviews to assess the feasibility and acceptability of the Snacktivity™ intervention to inform the design of a subsequent phase III randomised trial. A two-arm randomised controlled feasibility trial aiming to recruit 80 inactive adults will be conducted. Recruitment will be from health and community settings and social media. Participants will be individually randomised (1:1 ratio) to receive either the Snacktivity™ intervention or usual care. The intervention will last 12 weeks with assessment of outcomes completed before and after the intervention in all participants. We are interested in whether the Snacktivity™ trial is appealing to participants (assessed by the recruitment rate) and if the Snacktivity™ intervention and trial methods are acceptable to participants (assessed by Snacktivity™/physical activity adherence and retention rates). The intervention will be delivered by health care providers within health care consultations or by researchers. Participants’ experiences of the trial and intervention, and health care providers’ views of delivering the intervention within health consultations will be explored.

**Discussion:**

The development of physical activity interventions that can be delivered at scale are needed. The findings from this study will inform the viability and design of a phase III trial to assess the effectiveness and cost-effectiveness of Snacktivity™ to increase physical activity.

**Trial registration:**

ISRCTN: 64851242.

**Supplementary Information:**

The online version contains supplementary material available at 10.1186/s40814-023-01272-8.

## Background

Many people do not regularly participate in physical activity, which may adversely affect their health [1–3]. The prevention of non-communicable diseases is a major worldwide public health goal and improving lifestyle behaviours is considered essential to reducing the financial and health burden of non-communicable diseases [4]. Current physical activity guidelines are focused on weekly accumulation of at least 150 min of moderate to vigorous intensity physical activity per week (MVPA) [1]. This recommendation is often promoted as 30 min of MVPA on at least five days per week. Revised guidance now also recognises the importance of making small changes to physical activity behaviour, and that any physical activity is better than none. However, guidance still focuses on the public needing to achieve a behavioural goal of at least 150 min of moderate intensity physical activity per week [1, 5, 6]. Guidance also advises that adults should complete muscle and strength based physical activity on at least two days per week, but very few adults (~ 20%) achieve this regularly [7]. The low levels of participation in physical activity in the population is concerning and there is no reason to assume the situation will improve unless acceptable and effective interventions are put in place. Guidelines themselves do not change behaviour, it is having the means and motivation to achieve them that matters for public health.

### Snacktivity™

An alternative approach to physical activity promotion that could motivate the public to be more physically active throughout the whole day, is a concept referred to here as Snacktivity™ [8]. Rather than focusing on promoting 150 min of physical activity per week (e.g. ~ 30 min per day over 5 days), Snacktivity™ focuses on encouraging small, but frequent, doses of regular MVPA throughout the whole day such that at least 150 min of MVPA is accumulated weekly. A physical activity ‘snack’ lasts between 2 to 5 min, and examples include walk-talk conversations, walking coffee breaks, using stairs instead of the lift, pacing whilst using the telephone, or parking the car a little further away and walking to the destination. Of relevance here, updated guidance from health agencies around the world have removed the need for adults to complete physical activity in bouts lasting 10 min or more [1]. Improved cardio-metabolic aerobic fitness has been reported from brief bouts of physical activity [9–13], with other studies reporting no difference in the rate of improvement in cardiovascular fitness between accumulated and continuous bouts of physical activity lasting the same total duration [14].

How people feel about participation in physical activity is an important predictor of whether they will continue to engage and adhere with the activity [15]. A Snacktivity™ approach may help to develop individuals’ confidence to be physically active, particularly those who are inactive, by encouraging people to ‘start small’. Psychological theories recognise that achieving small changes is important for developing task and self-regulatory self-efficacy, as well as habit formation [16, 17]. Simple actions may become habitual more quickly than complex ones, suggesting that integration of small physical activity changes, or ‘snacks’, within everyday routines may be more feasible than longer ones, for the population to initiate and then sustain [16]. Thus, over time Snacktivity™ may be a vehicle for more sustained engagement in physical activity [8, 17]. Our earlier work has shown that the Snacktivity™ approach to promoting physical activity was viewed positively by the public [18, 19].

A common reason for inactivity is a perceived lack of time and Snacktivity™ provides an opportunity to address this barrier because it only requires a small amount of time, and no preparation/planning or equipment is required [20]. Snacktivity™ can also be incorporated into usual daily routines (e.g. getting off the bus a stop early on the way to work and walking meetings), and may therefore be perceived as appealing and feasible to achieve, compared with aiming for larger changes in physical activity. Snacktivity™ also provides the opportunity to promote the importance of muscle strength-based activities since many of these lend themselves to Snacktivity™ (e.g. squats when brushing your teeth and calf raises while waiting for the kettle to boil).

An inverse dose–response relationship exists between physical activity and all-cause mortality, therefore for people who are inactive any increase in physical activity is important [21, 22]. Snacktivity™ could also be important because the relationship between physical activity and mortality is also characterised by a steep early slope meaning the greatest gains in health are experienced when moving people who are inactive to 2–3 metabolic equivalent hours (MET/h) per week (~ 30 min per week), than moving more active people to doing marginally more [21–23]. In addition to concerns about low levels of participation in the population, there is growing concern about the amount of time the public spend in sedentary behaviours. Adults spend approximately 60–70% of their waking hours sedentary (e.g. sitting) [24]. This is of concern because too much time spent sedentary has been associated with type 2 diabetes, cardiovascular disease (CVD), all-cause and CVD-related mortality [25, 26], and guidelines now include recommendations about reducing time spent sedentary. An important additional benefit of Snacktivity™ is that it encourages breaking up sitting time throughout the day, meaning that it has the potential to impact two health behaviours simultaneously.

## Aims and objectives

Whilst the idea that small bouts of physical activity may improve health is not new, it is not a message that has been highlighted to the public, in part, because of a lack of robust evidence in real world health settings. To date, no randomised controlled trial (RCT) has directly investigated whether Snacktivity™ increases participation in MVPA or the number of people meeting the recommended guidelines for physical activity. The primary aim of the Snacktivity™ research programme is to evaluate the clinical and cost effectiveness of the Snacktivity™ intervention for increasing and maintaining physical activity by undertaking a large multicentre RCT. However, first we need to undertake a randomised feasibility trial with nested qualitative interviews to assess the feasibility and acceptability of the Snacktivity™ intervention and the methods needed to conduct the phase III randomised trial. We are seeking to test a complex intervention and there are uncertainties, specifically regarding recruitment methods and rates, intervention adherence and participant retention, that need to be assessed before undertaking a large-scale RCT.

This feasibility trial aims to:

- Examine the recruitment rate to inform the planned phase III trial.
- Provide data to review and inform the sample size assumptions for the phase III trial.
- Assess the data collection methods, data completeness and retention rates.
- Investigate the feasibility and acceptability of the Snacktivity™ intervention to the public and health care providers (HCPs) delivering the intervention.
- Assess adherence to the Snacktivity™ intervention
- Gather data to refine the Snacktivity™ intervention and assess intervention delivery fidelity.
- Examine the potential for intervention contamination, to inform the decision to use individual randomisation over a cluster RCT.
- Assess acceptability of the intervention training materials and procedures for HCPs.

## Methods

### Trial design and setting

**Fig. 1:**
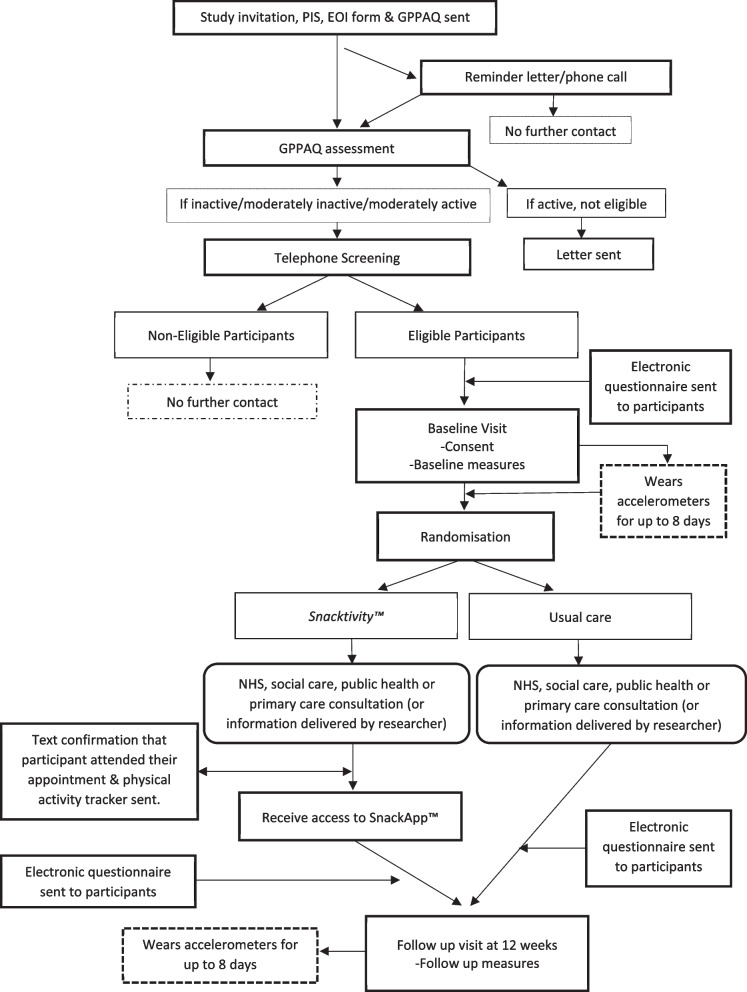
Participant flow

**Fig. 2:**
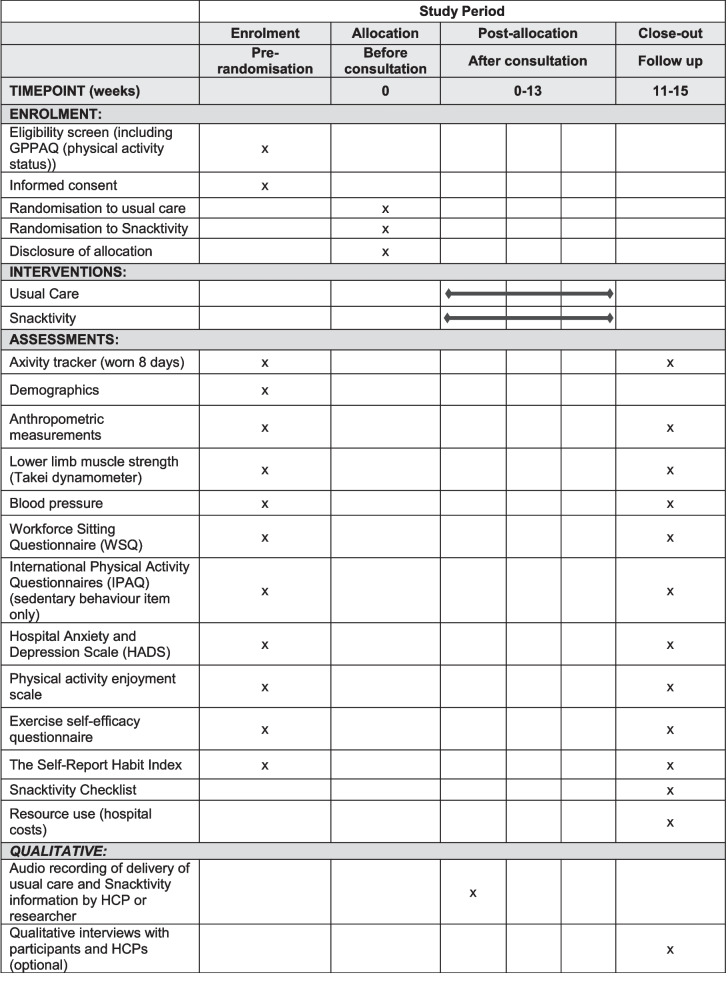
SPIRIT figure

A two-arm, multi-centre, individually randomised controlled feasibility trial will be conducted with a target recruitment of 80 participants across the East and West Midlands, UK. See Fig. 1 for participant flow. Recruitment will be from National Health Service (NHS), social care, public health, primary care services, community settings, and via social media. The trial will be conducted in accordance with the United Kingdom (UK) Policy Framework for Health and Social Care Research, the applicable UK Statutory Instruments, (which include the Data Protection Act 2018) and the principles of Good Clinical Practice. The study is following protocol version 7.0 (27 May 2022). Birmingham Community Healthcare NHS Foundation Trust in England are the sponsor for this trial. A SPIRIT Figure shows the different data collection steps of the trial (Fig. 2) and a completed SPIRIT checklist is available as an additional file (Additional file 1).

### Recruitment of participants

Participating general practices and NHS Trusts will search their electronic records to identify patients who are ≥ 18 years and have a health care consultation booked during the recruitment phase of the trial. These individuals will be sent a consultation appointment letter (or reminder of the upcoming appointment), along with the trial invitation letter, participant information sheet (PIS), expression of interest form (EOI) and the General Practice Physical Activity Questionnaire (GPPAQ) for screening physical activity status [27]. Those classed as inactive/moderately inactive/moderately active according to the GPPAQ (based on questions 1, 2a, and 2b) will be contacted by telephone to complete eligibility screening with a member of the research team. If a general practice or NHS Trust service routinely uses, or wishes to use, Short Message Service text messages to send patients details of their scheduled appointments and/or to notify them about this study, a study invite link will be added to these text messages where the trial documents referred to above can be accessed by those interested in taking part.

Clinicians/HCPs can also raise the topic of the Snacktivity™ study in routine consultations and those interested in discussing the trial further will be asked to complete the EOI form and GPPAQ, which will be passed on to the research team and these potential participants will complete eligibility screening with the research team by telephone. Potential participants can also be invited to take part in the study via community settings, including social media. These participants will either be sent the recruitment packs in the post or asked to complete the study documents electronically via the study invitation link as detailed above.

For all routes of recruitment, potentially eligible participants will be contacted by telephone by a member of the research team to complete further and final eligibility screening (see below for the full list of criteria applied). If eligible, a baseline visit will be booked to collect assessment data. For participants recruited via a reminder letter this will take place prior to participants’ health/intervention consultation appointment.

### Consent processes and steps

Potential participants will provide consent for all parts of the screening process described above, as well as consent to participate in the trial. Written informed consent for participation in the trial will be obtained for each participant by a researcher at the baseline visit. Where face to face visits are not possible, participants will be asked to provide written informed consent either online/email or by post. Participants will be made aware at the beginning of the study that they can freely withdraw (discontinue participation) from the trial (or part of) at any time without giving a reason.

### Participant eligibility

#### Inclusion

- Inactive, moderately inactive or moderately active (as measured by the GPPAQ) (27).
- Able to provide informed written consent.
- Aged ≥ 18 years.
- Own a mobile phone capable of hosting apps (Apple and Android).
- Agreement for their HCP to be notified of their involvement in the study (if applicable).

#### Exclusion criteria

- Unable to understand English sufficiently to complete the trial assessments.
- Women known to be pregnant or breast feeding.

#### Ineligible participants

If following screening an individual is not eligible, their identifiable data and contact details will be deleted from the trial system and any paper documents received from them will be destroyed. Verbal consent will be requested to store anonymous research data, for example, reasons why they were not eligible, to help inform any future trial.

### Randomisation and blinding

Once eligibility is confirmed, consent obtained and baseline data collected, participants will be randomised at the level of the individual in a 1:1 ratio to either the Snacktivity™ intervention or usual care. We have chosen individual rather than cluster randomisation for the definitive trial, and therefore also for this feasibility trial. An individually randomised trial also allows us to recruit participants outside a healthcare service/setting. Whilst there is a risk of contamination in an individually RCT, we anticipate the risk of contamination to be low and this will be assessed. Randomisation will be performed via a secure web-based service provided by the Birmingham Clinical Trials Unit. A minimisation algorithm will be used to ensure balance in the treatment allocation over the following variables: route of recruitment (primary care, community health service, other); age (18–45, ≥ 46 years); and gender (male, female). A ‘random element’ will be included in the minimisation algorithm. Participants are not informed of their group allocation until all the baseline data has been collected.

### Blinding

Participants will not be blinded to the exact purpose of the trial. It is not possible to blind the data collector, as the same researcher may be needed to undertake both the baseline and follow-up visit to collect data. We do not believe this will introduce bias, as the aim of this trial is to assess the feasibility of undertaking a large phase III RCT, and these outcomes are not affected by knowledge of group allocation and the data relating to the feasibility outcomes are not collected during the baseline and follow up visits. Only secondary outcomes are collected by researcher. The trial treatment allocation will be posted directly to the participants HCPs (where applicable). Physical activity will be assessed using a blinded research grade wrist worn accelerometer in both groups.

### The Snacktivity™ intervention

Participants randomised to the Snacktivity™ intervention and the current guidance for physical activity provided at NHS, social care, primary care or public health consultations will be advised to accumulate their physical activity through Snacktivity™. Snacktivity™ is defined as participation in small, but frequent, doses of regular MVPA throughout the day such that at least 150 min of MVPA is accumulated weekly. A physical activity ‘snack’ lasts between 2 to 5 min. The behavioural goal is for participants to work towards achieving at least 30 min of Snacktivity™ per day. The Snacktivity™ intervention aims to promote participation in Snacktivity™, the usefulness of Snacktivity™, encourages regular self-monitoring of Snacktivity™ to achieve sustained Snacktivity™, goal setting for daily Snacktivity™, as well as action planning and implementation strategies for Snacktivity™. There are two main components within the Snacktivity™ intervention; health professionals raising awareness of, and encouraging Snacktivity™ with participants in their consultations, and the promotion of technology to support behaviour change and sustained engagement in Snacktivity™ (via the phone app called SnackApp™ and physical activity self-monitoring device. See later for details.

The Snacktivity™ intervention is based on self-regulation theory and the habit formation model [28, 29]. Our own work and other studies have shown self-regulation/self-monitoring to be an effective foundation strategy for health behaviour change [30–32]. Self-monitoring of Snacktivity™ may act as a reward for individuals who increase their physical activity behaviour, who are then provided with positive feedback from the monitoring process, thereby enhancing their motivation and reducing the potential for relapse. Frequent monitoring and reflection of Snacktivity™ progress may also improve self-efficacy for participation in both short and longer bouts of physical activity. Encouragement of self-monitoring and recording of Snacktivity™ is a simple concept for a health professional to advocate as a public health communication. It is simple for people to understand and implement. Trials have shown that participants can adhere to daily self-monitoring of physical activity [33]. An intervention logic model was developed to guide the content and nature of the intervention.

### Snacktivity™ consultation

Participants will receive standard guidance about the importance of physical activity and any behavioural change strategies usually adopted by HCPs, but they will be advised to accumulate their physical activity through Snacktivity™. The intervention will start with delivery of the Snacktivity™ message. HCPs will also briefly discuss the purpose of Snacktivity™, the hypothesis underpinning its principals, how it differs from standard physical activity advice, and provide examples of Snacktivity™. HCP’s will use a picture board that illustrates a range of activity snacks.

HCPs will promote the rationale for Snacktivity™ and the benefits of physical activity for health, give examples of Snacktivity™, explain implementation plans and action planning. HCPs will highlight to participants that Snacktivity™ will work best if they develop habits or routines and how participants can achieve this. The purpose of the physical activity monitor and SnackApp™ for facilitating self-monitoring of activity snacks will be discussed; use of this technology will be specifically encouraged. An intervention checklist will be completed by HCPs to aid delivery and provide a reminder prompt of areas that must be covered during consultations. We anticipate the delivery of the Snacktivity™ intervention to participants taking approximately 5 to 7 min. The role of the HCP is to simply raise the topic of Snacktivity™/physical activity, to signpost participants to the SnackApp™ for further advice and support, and to encourage participants to use their physical activity monitoring device to facilitate their engagement with Snacktivity™ and to obtain feedback.

If a participant is recruited via community settings, including third party organisations and social media, a researcher will call the participant and deliver the intervention over the telephone. The consultation will be delivered following the same protocol and intervention checklist as is being used by HCPs. The delivery of the intervention by both HCPs and researchers will be audio recorded to assess fidelity (only if the participant has consented to this).

### SnackApp™ and physical activity monitoring device

As part of the intervention participants receive access to the mobile phone application SnackApp™ and are provided with a physical activity monitoring device (Fitbit). Technology-based interventions offer some key advantages over traditional behaviour change interventions and have the potential to reach a large number of people at a relatively low cost and offers increased access to the public at a time and place that suits their preferences, including the ability to overcome the need to attend face-to-face sessions to receive the intervention. As a population approach, smartphone-based interventions are very attractive as 90% of mobile phone users are in possession of their telephones 24 h per day. mHealth technology is applicable across the age and cultural spectrums and studies have shown that electronic devices that promote physical activity are acceptable [34, 35]. In earlier work to survey the views of the public about the Snacktivity™ concept, and the use of technology to support Snacktivity™, 90% of 724 respondents were found to own a smartphone and 45% used their phone to monitor their physical activity [17].

To facilitate habit formation the SnackApp™ will generate regular reminders and notifications for the intervention group to engage in Snacktivity™. Self-monitoring may be particularly relevant for developing Snacktivity™ habits because it may be more difficult for people to easily recall how many activity snacks they have achieved each day. The design and content of the SnackApp™ is based on previous work packages, some of the principals of existing apps, and our previous experiences of developing apps for promoting physical activity and lifestyle behaviours. Participants will receive free access to the SnackApp™ after their consultation, which, within its functions, will contain features common in digital health interventions. Within these functions, the SnackApp™:

- Automatically captures/monitors daily physical activity and inactive time via a wrist worn consumer monitor (Fitbit Versa 2 device). It provides measures of steps, activity level, inactivity and energy expenditure each minute. A companion SnackApp™ will be downloaded onto the clock face of the Versa to allow instantaneous feedback on Snacktivity™ progress throughout the intervention period.
- Classifies participants into an activity profile according to their active and inactive time.
- Provides physical activity prompts after user-defined inactive periods, encouraging regular active snacks throughout the day.
- Generates individualised motivational push notifications to participants’ mobile phones based on prior behaviour and supports goal setting (both automatic and user-defined).
- Provides individualised feedback related to goal achievement to encourage adherence and facilitate self-efficacy. Individualised feedback will be provided on the accumulated number of physical activity snacks completed and total minutes ‘activity snacking’ per day, progress towards meeting the recommended level of physical activity for health benefits (150 min of MVPA per week) and the total number of minutes of physical activity, MVPA and the number of steps each day.
- Enables access to educational content (text, static images, video, and audio content) regarding Snacktivity™, both externally hosted and supplied by the research team, including examples of activity snacks (with instructive photos and gifs)
- Encourages social support via a forum to facilitate a Snacktivity™ social community.
- To promote habit formation, users will be able to plan when to perform activity snacks.

### Training of HCPs/researchers to deliver the Snacktivity™ intervention

Those delivering the intervention will be trained by the research team to deliver the Snacktivity™ intervention following a standard protocol; we have developed a media-based training module that can be delivered face-to-face or remotely. We anticipate the training will take no more than 1 h given the involvement of HCPs/researchers is simple and brief. The training tools will include information on the importance of adhering to the study protocol, the research study procedures, trial design and ways of delivering the Snacktivity™ intervention. More specifically, the training aims to demonstrate different ways in which Snacktivity*™* can be promoted by HCPs within the consultation (where appropriate). We have also developed video clips that show Snacktivity™ being delivered in GP practices, so that HCPs have an understanding of how the intervention might be delivered by them.

### Intervention fidelity and contamination

With the consent of participants, the delivery of the Snacktivity™ information will be audio-recorded to assess for fidelity against the intervention component checklist. Those delivering the intervention will be trained on the importance of delivering the correct information to participants according to their randomised group to minimise the possibility of contamination. The Fitbit device and SnackApp will only be available to participants randomised to the intervention group. A range of the strategies to reinforce intervention fidelity will be used. We will.

- Develop a standardised training resource.
- Train HCPs/researchers to deliver the intervention according to the protocol.
- Explain the intervention logic model to HCPs/researchers.
- Audio record all consultations and telephone calls with a researcher to assess whether the intervention is being delivered according to the intervention checklist (with participant consent).
- Check for intervention ‘receipt’ and enactment by checking whether participants attended their consultation and whether the physical activity self-monitoring device and SnackApp™ are activated.

### Comparator group

We are not proposing any change to standard care. The usual care group will therefore receive the normal physical activity advice and any typical behavioural change strategies adopted by HCPs within consultations. The usual care group will only receive the current guidance for physical activity (to achieve 150 min of MVPA) within their healthcare consultation or during the telephone call with a researcher, who will advise they work towards the accumulation of at least 150 min MVPA per week. Participants will also receive a leaflet. With the consent of participants, the delivery of the usual care information will be audio-recorded to assess fidelity and intervention contamination.

### Primary outcome

The primary outcome is the feasibility and acceptability of a subsequent phase III RCT according to pre-specified progression criteria. We are primarily interested in whether the Snacktivity™ intervention and trial are appealing to participants (assessed by the recruitment rate) and if the Snacktivity™ intervention and the evaluation methods are acceptable to participants (measured by Snacktivity™/physical activity adherence and retention rates). We also wish to assess the recruitment and randomisation processes, measure the extent of any intervention contamination, and use data collected in the feasibility trial to review the sample size assumptions for the phase III trial.

### Progression criteria for phase III trial and stop–go criteria

The decision of whether to continue to the phase III trial will be guided by the assessment of the data collected during the feasibility trial (both quantitative and qualitative). For the quantitative data (Table 1), the following pre-defined stop–go criteria will be used:

- *Recruitment*: defined as the number/percentage of people randomised against the recruitment target of 80 participants over 5 months.
- Snacktivity*™ adherence*: defined as the number of physical activity snacks achieved (defined as minimum of four bouts of MVPA lasting ≥ 2 min on average each day over 12 weeks) assessed by the Fitbit monitoring device (in the Snacktivity*™* arm only).
- *Physical activity adherence*: defined as the proportion of participants who are accumulating a total weekly average of at least 105 min of MVPA (~ 15 min daily) (in the Snacktivity*™* arm only).
- *Attrition*: defined as withdrawal from the trial and/or no follow-up data available.

**Table 1:** Traffic light criteria

Green	At least 80% of the target sample size is recruited
	At least 65% of the intervention group are achieving Snacktivity™ adherence
	At least 60% of the intervention group are achieving physical activity adherence
	Attrition < 21%
If all four criteria are met, we will proceed to the full trial with the protocol unchanged (unless there is a clear indication from the qualitative interviews and our experience that would improve the protocol)	
Amber	50–79% of the target sample size is recruited
	45–64% of the intervention group are achieving Snacktivity™ adherence
	45–59% of the intervention group are achieving physical activity adherence
	Attrition 21–35%
If one or more of our amber criteria are met, we will plan to adapt the protocol in light of the results of the feedback from the qualitative interviews and our experience to improve which ever criteria are not at the ‘green light’ level before proceeding to the full trial	
Red	< 50% of the target sample size is recruited
	< 45% of the intervention group are achieving Snacktivity™ adherence
	< 45% of the intervention group are achieving physical activity adherence
	Attrition > 35%
If one or more of these criteria are met, we would consider the current protocol not feasible and not progress to the phase III RCT with the current protocol	

### Secondary outcomes

Data will be collected on outcomes that we plan to collect in the definitive RCT at baseline and follow-up (see Table 2). While this feasibility trial is not powered to detect meaningful differences in these outcomes, collecting this data means we can assess and ensure that there are no issues with the collection and completion of these measures in preparation for the phase III trial. We will calculate MVPA, total physical activity, light physical activity, sedentary time (i.e. inactive time during waking hours), and sleep (and other metrics that may become available through novel processing methods) using data collected from a wrist worn research grade blinded accelerometer on participants’ non-dominant wrist (Axivity AX3; Axivity, Newcastle, UK) for at least seven days in both groups. A self-reported wake and sleep times log will be completed during the same days the accelerometer is worn. Other data collected at baseline and follow-up in all participants include self-reported sedentary behaviours (using the Workforce Sitting Questionnaire (WSQ) [36] and the sedentary behaviour item from the International Physical Activity Questionnaire [37], lower limb muscle strength (Takei dynamometer squat position), weight, waist circumference, blood pressure, and depression/anxiety (Hospital Anxiety and Depression Scale) [38], Physical Activity Enjoyment Scale [39] and Exercise Self-efficacy Questionnaire [40]. The Self-Report Habit Index [41] and a checklist of popular activity snacks questionnaire (Snacktivity™ checklist) are completed at follow-up in the intervention group only. Participants’ experiences of the trial will be captured in single item exit questions at follow-up, which includes questions relating to contamination. Specifically, participants are asked whether they know anyone else taking part in the study, and if so, whether they discussed the study with them.

**Table 2:** Schedule of assessments

Visit	Screening	Baseline visit (− 7 + 14 days)	Follow-up (12 weeks) (− or + 14 days)
Expression of interest	x		
Physical activity status: General Practice Physical Activity Questionnaire) [27]	x		
Eligibility screening telephone call	x		
Personal identifiers and demographic information		x	
Current medications			x
Smoking history		x	
Alcohol consumption		x	
Mobility		x	
Diseases/conditions		x	x
Wrist worn accelerometer (worn for up to 8 days) (axivity)		x	x
Anxiety and depression: HADS [38]		x	x
Healthcare utilisation			x
Household income/composition			x
Productivity			x
Sedentary behaviours: Workforce Sitting Questionnaire [36] and sedentary behaviour item from the International Physical Activity Questionnaire [37]		x	x
Enjoyment of physical activity: Physical Activity Enjoyment Scale [39]		x	x
Self-efficacy for exercise and Exercise Self-efficacy Questionnaire [40]		x	x
Habit strength (Snacktivity*™* group only): The Self-Report Habit Index [41]			x
SnackApp™ engagement analytics (Snacktivity*™* group only)			x
Height^a^		x	
Weight		x	x
Body mass index (BMI)		x	x
Waist circumference		x	x
Lower limb muscle strength (Takei dynamometer squat position)		x	x
Blood pressure		x	x
Checklist of popular snacks (paper copy, Snacktivity*™* group only)			x
Semi-structured interviews (Snacktivity*™* intervention group and health care providers)		x	x
Single item study feedback questions			x

^a^To allow for the calculation of BMI, height will be measured to the nearest 0.1 cm using SECA 213 stadiometers at baseline and follow-up

We plan to conduct a cost effectiveness study in the subsequent phase III trial and the questionnaire items to be used to assess health care resource use and productivity will be piloted in this feasibility trial. The physical activity self-monitoring device (Fitbit) will provide the following data for participants in the intervention group; steps, distance, calories, bouted active minutes, inactive time, sleep and awake time, and wear time (through body sensor). The Snacktivity™ active minutes will be computed from Fitbit device measured METs and activity snacks.

### Data collection

At baseline participants wear the axivity accelerometer for at least seven days before randomisation and complete the baseline questionnaires (either using an online link or paper copy sent by post) before the baseline home visit. At follow-up, the accelerometer is posted to participants in advance of their follow up visit, and the questionnaires are sent to participants once the follow-up visit has been booked (online link or paper as described above). The study visits will be conducted face to face by a member of the research team at participants’ home, a community venue or at their GP practice and completed in line with Government COVID-19 guidance, including NHS infection control procedures. If in person data collection cannot be completed, assessments will take place remotely online or using video conferencing tools. All data collected from participants is stored in a secure password protected database. All participants will receive a £20 high street voucher at completion of follow-up. All data is collected according to a data management plan.

### Collection of SnackApp^TM^ data

**Fig. 3:**
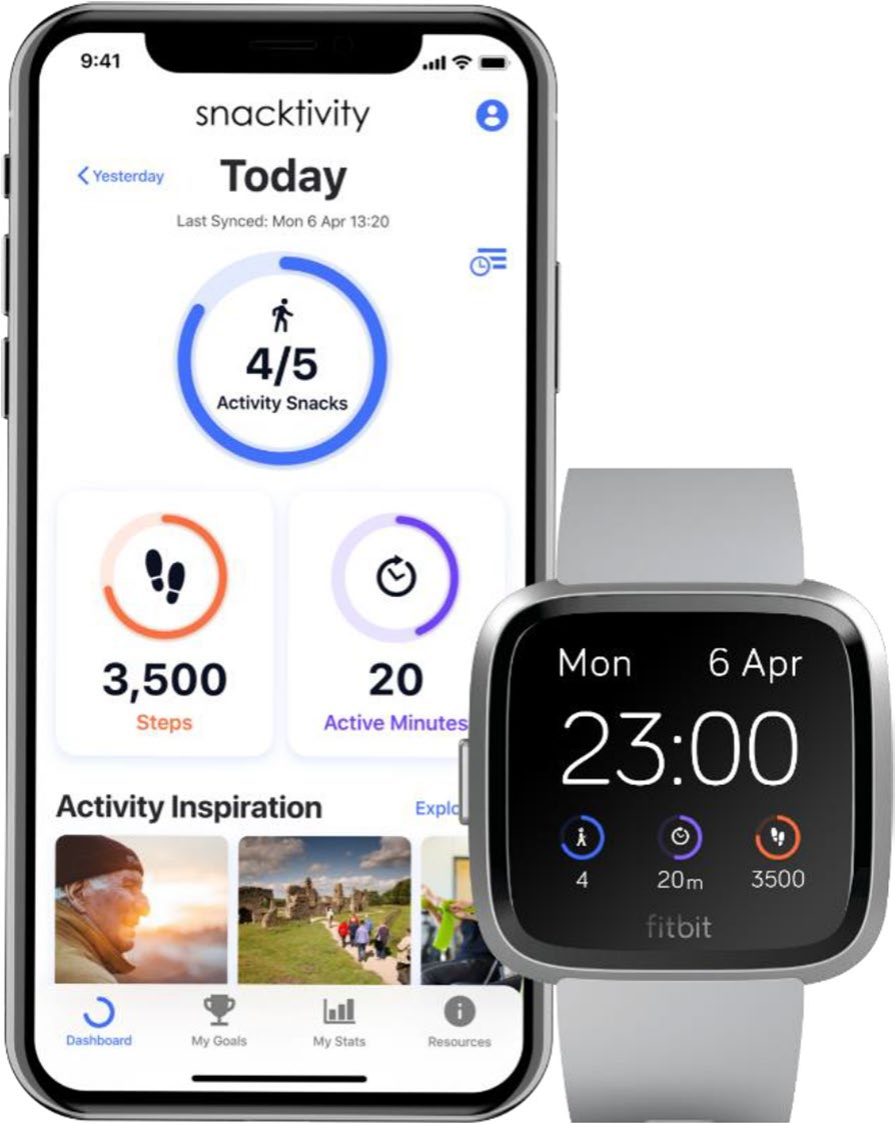
SnackApp^TM^ clockface

The Fitbit Versa 2 collects various measures of physical activity data and displays these data using a bespoke SnackApp^TM^ clockface (see Fig. 3). The SnackApp^TM^ clockface, which is set as default, provides immediate feedback on the number of activity snacks, ‘active minutes’ (i.e. MVPA) and the number of steps users have achieved as measured by Fitbit Versa 2 watch. The clockface on the Fitbit smartwatch downloads the data to a ‘companion app’ via Bluetooth low energy which sit within the Fitbit app environment. With an internet connection, the companion app uploads the data to the Snacktivity™ application programming interface (API) where they are stored in PostgreSQL databases hosted on a secure encrypted Google server. Engagement analytic from the SnackApp^TM^ will be collected and defined as participants’ use and interactions with the SnackApp^TM^ and Fitbit clockface.

### Adverse event reporting

There is no reason to assume that this trial will lead to an excess of adverse events. The intervention consists of HCPs/researchers promoting short bouts of MVPA within everyday life and use of a commercially available physical activity device to monitor activity, along with a mobile phone app to log movement, none of which are likely to create harm. Furthermore, the promotion of physical activity by health providers is already part of standard care and has been demonstrated as being low risk for all citizens in England as per the NHS Making Every Contact Count Campaign [42] without specific follow-up for adverse events. Therefore, no adverse events will be collected.

### Serious adverse events (SAE)

The research team at site (where applicable) will report all SAEs that are not defined as protocol exempt in an expedited manner. The following are ‘protocol exempt’ SAEs: events related to the participants pre-existing condition(s) (pre-existing conditions are medical conditions that existed before entering the trial, or for which they have already consulted medical advice, as identified on the baseline questionnaire (as per the diseases/conditions specified in Table 3)); and hospital visits for any elective procedures. Musculoskeletal and bone injuries/fractures, and trips and fall injuries are regarded as expected SAEs and are recorded on the follow-up case report forms.

**Table 3:** Protocol exempt SAEs relating to pre-existing conditions

Cancer	Sarcopenia (loss of muscle strength)
Type 1 diabetes	Chronic obstructive pulmonary disease
Type 2 diabetes	Asthma
High cholesterol	Kidney disease
High blood pressure	Back pain resulting in time off work
Heart disease, heart attack, angina, aneurysm	Rheumatoid arthritis
Stroke	Osteoarthritis
Depression or anxiety	Neurological condition (e.g. epilepsy, myalgic encephalomyelitis, or multiple sclerosis
Dementia or Alzheimer’s disease	COVID-19
Osteoporosis	Foot/ankle problem affecting patient’s mobility
Obesity	

### Statistical considerations and data analysis

The feasibility trial aims to recruit 80 participants over 5 months and the sample size is based on recommended sample sizes for feasibility and pilot trials [43]. This means that we will be able to estimate a Snacktivity™ adherence rate of 65% to within a 95% confidence interval of ± 14.8% (based on *n* = 40); a physical activity adherence rate of 60% to within a 95% confidence interval of ± 15.2% (based on *n* = 40); and an attrition rate of 20% to within a 95% confidence interval of ± 8.8% (based on *n* = 80). Based on a response rate of between 1 and 2%, 4000 to 5000 people will need to be invited to participate in the trial to achieve the required number of participants.

A separate statistical analysis plan will provide a more comprehensive description of the planned statistical analyses. The data analysis for this feasibility trial will be mainly descriptive, and focus on confidence interval estimation, with no hypothesis testing performed and no p-values presented. A brief outline of the planned analyses in relation to the stop–go criteria for this feasibility trial is provided. Recruitment and attrition rates will be analysed by pooling the two randomised groups. Adherence (Snacktivity™ and physical activity) rates will be assessed for the Snacktivity*™* group only. Progression criteria will be summarised as proportions and percentages with 95% confidence intervals. We will collect data on the number of invitations sent, and aggregated data on the age and gender of invitees to compare with the trial participants.

### Interview study

To gain further insight into the process of intervention delivery and receipt and the Snacktivity™ intervention we will ask participants to complete semi structured interviews about their experiences of the Snacktivity™ intervention. Questions informed by self-regulation and the habit formation model will focus on how often and how easy activity snacking is to do, how many snacks are achievable, ideal timing for snacking, barriers to snacking, formation of habits and implementation strategies used. Feedback on the use of the SnackApp™ and the suggested activity snacks will also be collected. Questions will also be guided by the intervention logic model. We expect to complete 20–25 interviews which should allow for saturation to be reached, as recommended for this type of study [44]. Purposive sampling will enable inclusion of participants who reflect as many socio-demographic characteristics of the possible eligible population (e.g. age, gender, ethnicity, socio-economic status, general exercise behaviour), and display different levels of engagement and participation with Snacktivity to capture the range of views.

Interviews will be audio-recorded, transcribed verbatim and analysed using a framework approach [45]. As well as documenting individual and overall themes we will carry out theme comparison as appropriate, for example across socio-demographic characteristics and engagement level. Findings will be interpreted against and combined with other study data. This will help us to understand the intervention process and the participants’ experiences, allowing us to further refine the intervention as necessary [46].

HCPs delivering the intervention will also be interviewed to gain their views and feedback about delivering the Snacktivity™ intervention within their routine consultations. We anticipate conducting 12–15 interviews with a range of HCPs. Interviews will be recorded, transcribed and analysed. Data will be interpreted as described above by means of overall and individual themes, theme comparison and in the context of findings from participant interviews and other study findings. Data will be used to inform and maximise participants experiences of receiving the Snacktivity intervention and HCPs experience of delivering the intervention in the Phase III RCT.

### Trial oversight and management

The Trial Management Group, which includes two members of the patient advisory group, will meet regularly (approximately every month) to ensure successful implementation and delivery of the trial. They will monitor participant recruitment; any departure from the expected recruitment rate will be dealt with according to the specific issues that arise. A joint independent Trial Steering Committee and Data Monitoring Committee (TSC/DMC) has been created for the Snacktivity™ feasibility trial. The TSC/DMC will meet at least twice a year or as required depending on the needs of the trial. The joint TSC/DMC will provide overall oversight of the trial, including the practical aspects of the trial, as well as ensuring that the trial is run in a way which is both safe for the participants and provides appropriate feasibility data to the sponsor and investigators.

### Public and patient involvement

This trial is supported by a Public Advisory Group (PAG) that consists of 10 members of the public from a range of backgrounds with different attitudes towards physical activity. The PAG is facilitated by a public involvement Lead and PAG members contribute to the development of the Snacktivity intervention through attending quarterly meetings or completing tasks remotely (e.g. providing feedback on documentation). Progress updates will be provided in periods between meetings (either short online meetings or newsletter). The PAG will have input into the design and development of the phase III trial and will be invited to comment on all participant facing documents and strategies for recruitment. Two PAG members will be part of the Trial Management Group and one PAG member will be attend the Trial Steering Group meetings. All PAG members will be offered an honoraria for involvement, which aligns to the National Institute for Health and Care Research (NIHR) Centre for Engagement and Dissemination recommendations.

### Trial progress

The trial has so far recruited 72 participants who have completed their baseline assessment and follow-up is ongoing. Recruitment to the qualitative study is ongoing and expected to be completed in February 2023.

## Discussion

There is strong evidence that physical inactivity and high levels of sitting are associated with poorer health and mortality, yet the population has become less physically active. Interventions that can be delivered at scale to address these health behaviours are required. In previous research, we reported that the Snacktivity™ approach to promoting physical activity was viewed positively by the public [17, 18]. In this research, we are proposing that Snacktivity™ may be an alternative way of promoting participation in physical activity to the public and assessing whether a full-scale trial is feasible. While there might be advantages to the Snacktivity™ approach, there may also be disadvantages. Snacktivity™ may be disruptive to the day, it may be easily forgotten, or difficult to achieve MVPA in short bouts. It may also not impact health sufficiently, or it may be difficult for the public to think of ways to achieve Snacktivity™ or implement it into their everyday lives. Research needs to explore these possible issues and consider how any potential barriers to Snacktivity™ might be overcome and this trial will allow us to assess and understand any issues before embarking on a subsequent phase III trial. Using both quantitative and qualitative methods the results of this trial will provide robust evidence regarding the feasibility and acceptability of an alternative approach to promoting physical activity in the population.

## Supplementary Information

**Additional file 1.** SPIRIT 2013 Checklist: Recommended items to address in a clinical trial protocol and related documents*.

## Data Availability

Data sharing is not applicable to this article as no datasets were generated or analysed during the current study.

## Abbreviations

API: Application Programming Interface
ARC: Applied Research Collaboration
BMI: Body mass index
CRF: Case Report Forms
CVD: Cardiovascular disease
DMC: Data Monitoring Committee
EOI: Expression of Interest Form
GPPAQ: General Practice Physical Activity Questionnaire
HADS: Hospital Anxiety and Depression Scale
HCP: Health care provider
IPAQ: International Physical Activity Questionnaire
METs: Metabolic equivalents
MVPA: Moderate to Vigorous Intensity Physical Activity
NIHR: National Institute for Health and Care Research
NHS: National Health Service
PAG: Patient Advisory Group
PIS: Participant Information Sheet
RCT: Randomised controlled trial
SAE: Serious adverse event
TSC: Trial Steering Committee
UK: United Kingdom
WSQ: Workforce Sitting Questionnaire
